# Application of network pharmacology and dock of molecules on the exploration of the mechanism of frankincense-myrrh for lumbar intervertebral disc degeneration: A review

**DOI:** 10.1097/MD.0000000000038953

**Published:** 2024-07-19

**Authors:** Yun Lu, Haopeng Luan, Cong Peng, Junjie Ma, Zhe Li, Yu Hu, Xinghua Song

**Affiliations:** a Department of Spine Surgery, The Sixth Affiliated Hospital of Xinjiang Medical University, Urumqi, Xinjiang, 830002, China; b Department of Pharmacy, The Sixth Affiliated Hospital of Xinjiang Medical University, Urumqi, Xinjiang, China.

**Keywords:** dock of molecules, frankincense-myrrh, lumbar intervertebral disc degeneration, network pharmacology

## Abstract

To investigate the efficacy of Frankincense-Myrrh in lumbar Intervertebral degenerative diseases (LIDD). The active components of frankincense-myrrh was retrieved with a unique system pharmacology platform for Traditional Chinese Medicine Systems Pharmacology (TCMSP). The LIDD-related target genes were screened with DisGeNET and Genecards databases. Then, STRING & Cytoscape were used for analyzing the Protein-Protein Interaction network. DAVID was used for analyzing Gene Ontology (GO) & Kyoto Encyclopedia of Genes and Genomes (KEGG) enrichment. Finally, molecules of AutoDockVina and Pymol were used for docking the molecules for verifying active ingredients and key targets’ binding force. The 105 LIDD-related targets identified in Ruxiang (RX)-Moyao (MY) involve 53 active ingredients. In addition, topological analysis was conducted for identifying the 12 key targets. According to the analysis results of GO & KEGG, RX-MY is significant for treating LIDD through participating in many pathways and biological processes, such as signaling pathways of inflammatory response reactive process, MAP kinase activity, TNF, and MAPK, etc. According to the dock results, the active components oxo-tirucalic, acid, isofouquierone, (7S, 8R, 9S, 10R, 13S, 14S,17Z)-17-ethylidene-7-hydroxy-10,13-dimethyl-1,2,6,7,8,9,11,12,14,15-decahydrocyclopenta [a] phenanthrene-3,16-dion in RX-MY binds actively. The basic pharmacological action and RX-MY-related mechanism in the treatment of LIDD was revealed in this study for the first time. It is predicted that the results may provide a treatment plan for RX-MY with replacement of NSAIDs and warrant investigation of new therapeutic alternatives for LIDD. However, these predictions should be validated by relevant pharmacological trials.

## 1. Introduction

As global aging, back pain issues in the elderly attract global attention.^[1,2]^ Low back pain is mainly caused by lumbar intervertebral disc degeneration (LIDD),^[3]^ which is a chronic, common and frequently-occurring disease characterized by degenerative pathological changes, often occurring in the elderly. It is with the clinical characteristics of delayed and refractory, high recurrence rate and disability rates. In 2013, 16.347 million people died in China due to disability for low back pain, occupying the most disability burden of Chinese people, resulting in a great economic burden and mental pressure to people.^[4]^ LIDD is the core link of this disease, which pathological mechanism involves many complex biological processes, including stimulation of inflammation and immunoreaction, and cell apoptosis.^[5–7]^ Clinically, the ideal treatment of LIDD shall realize at least 1 of the 3 objectives as follows: relieving back pain, reducing or reversing the metabolism of intervertebral disc degeneration (IVDD) tissues or promoting the growth of tissues. Through conforming several studies, effective ingredients in Chinese medicine can inhibit LIDD and promote the regeneration of tissues.^[8,9]^ According to the study with the V2.5 software of traditional Chinese medicine (TCM) inheritance assistant platform, “frankincense-myrrh” is one of the core drug combinations^[10]^ for treating lumbar disc herniation.

As 2 different olive plants, Ruxiang (RX)-Moyao (MY) as well as their gelly-like resins secreted when their trunks are cut have medicinal properties,^[11]^ which are usually applied to the clinical improvement of blood circulation and elimination of blood stasis, and easing pain and generating muscles. Furthermore, drug combination is also used with better inflammation resistance and analgesic effects on the peripheral and nervous centralises in mice with acute inflammatory pain caused by tail flick in a hot water bath and writhing in acetic acid,^[12,13]^ and has strong inflammation resistance clinical effect.^[14]^ In TCM, drug combination has better effectiveness and safety than that of single herbal therapy, because it produces synergistic effects and neutralizes potential toxicities in interaction process.^[15]^

**Figure 1.**
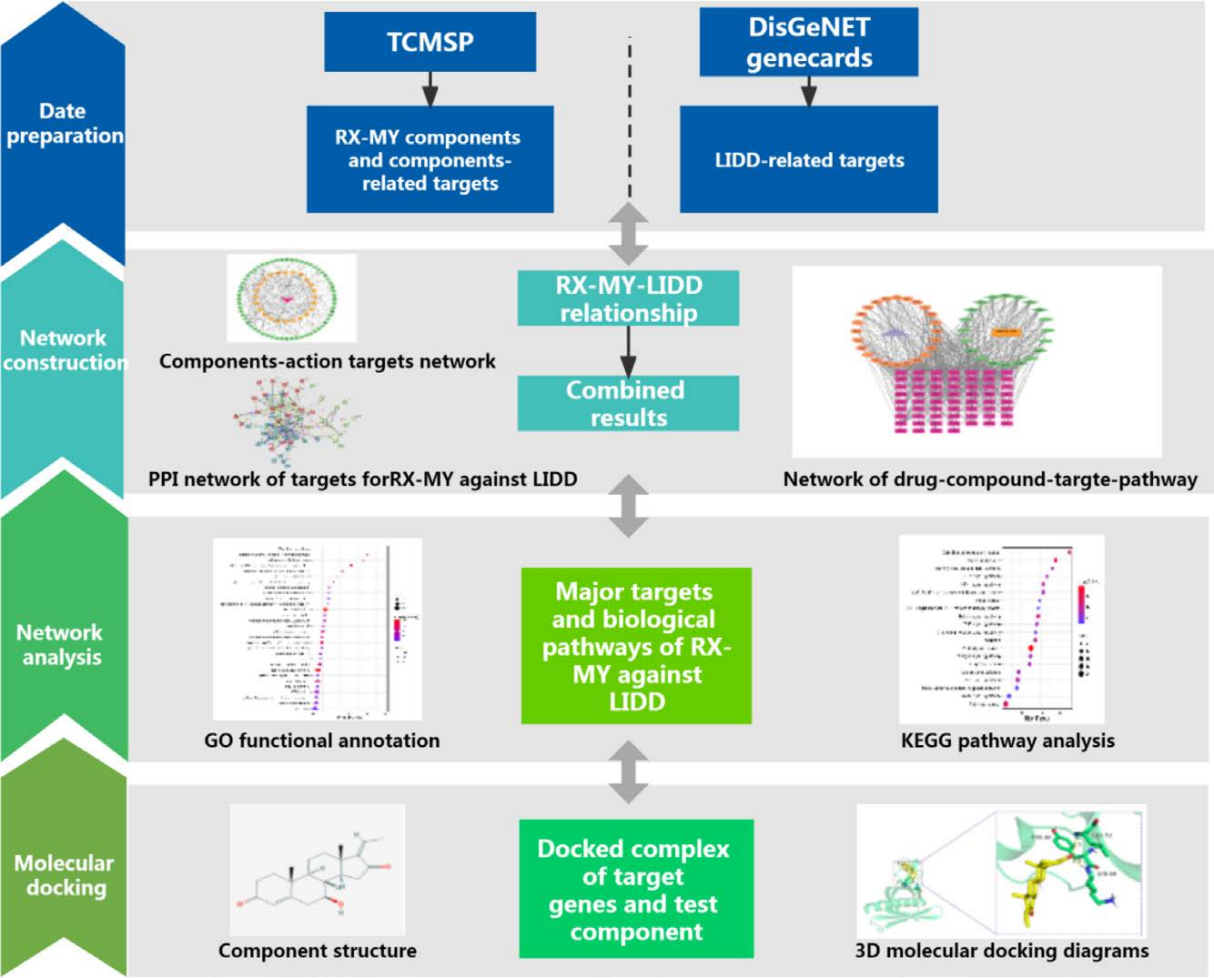
Workflow for treating LIDD with RX-MY. LIDD = lumbar intervertebral disc degeneration, MY = Moyao, RX = Ruxiang.

Network pharmacology WAS deeply analyzed with high-throughput omics data, etc. A holistic and systematic method WAS used herein based on the overall concept and dialectical principles of TCM. The inflammation resistance and analgesic properties of frankincense and myrrh, and their mechanism for improving lumbar degenerative disease are still unclear. The treating mechanism of RX-MY for treating LIDD through combined pharmacology and molecular docking was preliminarily discussed, which founded a basis for further studying and clinical use theoretically, which workflow is shown in Figure 1.

## 2. Method

### 2.1. Bioactive compounds of RX-MY

Traditional Chinese Medicine Systems Pharmacology (TCMSP) was used for collecting all the compounds of frankincense - myrrh. According to previous reports, the parameters of ADME as well as other pharmacokinetics significantly affect the bioactivities of drugs. According to the recommendation of TCMSP, oral taking the compounds with 30% OB and high absorption and low metabolism. Compounds with 0:18 DL were chemically applicable for developing drugs. Therefore, 2 adme-related parameters were used for identifying potentially active compounds in RX-MY, i.e. OB and DL are ≥ 30% and ≥ 0:18, respectively. If OB and DL ≥ 30% and ≥ 0:18, respectively, the most active compounds of RX-MY were identified.

### 2.2. Acquisition of main components and disease-related genes

PubChem was applied for obtaining the potential active ingredients’ chemical structure, which database RX-MY was constructed. The SMILES of the main compounds in frankincense-myrrh retrieved were imported into the SwissTargetPrediction database^[16,17]^ for generating the gene name (protein name) of a single main component, and screening the possible target with probability > 0.

### 2.3. Identifying LIDD-related targets and its predicting therapeutic targets

The GeneCards and DisGeNET databases were searched for detailed information about mankind genes related to lumbar degenerative diseases. Only “Homo sapiens” genes linked to LIDD with the keyword “Degeneration of lumbar intervertebral disc” were acquired, combining full target genes from the foresaid 2 databases and deleted duplicated genes. Thus, active compound targets were mapped to those correlated to LIDD, and RX-MY targets for treating LIDD with the Venny 2.1.

### 2.4. PPI data

In the STRING database, there are expected and experimental protein interactions. Target effect was inputted into the STRING database as symbols for genes, limited to “Homo sapiens,” with hidden free points. The confidence score > 0.7, which was high for generating Protein-Protein Interaction (PPI) data and saving the tsv file for future study.

### 2.5. Structure analysis

The following steps were taken to construct the network: the RX-MY decoction compound network was formed through the connection between RX-MY with corresponding active compounds; RX-MY has created an active network targeting on compounds through connecting active compounds to their targets; An RX-MY therapeutic target network against LIDD was constructed; A topological analysis was conducted to identify key targets for RX-MY against LIDD; A network was constructed, which were visualized with V3.9.1 Cytoscape, which is helpful for analyzing and visualizing molecular interactions.

Plug-in of Cytoscape, a CytoNCA tool was used for analyzing targets’ topological properties, which key genes were identified with high connectivity for treating LIDD with RX-MY. Four elements were used for estimating the core properties of the network nodes, which are represented with DC, BC, CC, and EC, respectively. Nodes with higher 4 quantitative values are more significant for the network. DC, BC, CC, and EC in PPI are higher than their respective average values for screening the key targets of RX-MY on LIDD.

### 2.6. Annotation of GO functional and analysis of KEGG pathway

The enrichment was analyzed with DAVID, and the protein groups existing in the gene ontology (GO) notes were evaluated quantitatively by statistical hypergeometric distribution (*P* value),^[18]^ reflecting the significance of protein biological function with *P* value. It was used for enriching and analyzing the Kyoto Encyclopedia of Genes and Genomes (KEGG) pathway of the intersection protein target gene between drug and disease, and the main action pathway of RX-M used for treating LIDD was obtained. Functionally annotated and enriched key target proteins shared by RX-M and LIDD were analyzed, and the target and related signaling pathways were explored, which are represented with CC, MF, BP, and KEGG, respectively. Finally, a bioinformatics website was used to draw a bubble chart for visualization.

### 2.7. Component-target molecular docking

The specific operation is as follows: As a small molecule-ligand, the key active ingredient downloads the 3D structure in MOL2 format from the TCMSP database according to the small-molecule MOLID, imports the structure into ChemBio3D Ultra 14.0, and saves it in mol2 format. The core target was used as the receptor and the top 5 of DC were selected as target proteins. Through the PBD database, the 3D structure was downloaded (the core target protein follows: human protein, resolution, and preference have the original ligand) in PDB form. Protein crystal water, original ligand, etc were removed with V 2.3.0 Pymol, and then the protein structure was imported into V1.5.6 AutoDock for hydrogenating, calculating and distributing charge, and designating atom type, and saved as “pdbqt” format. V1.1 POCASA was used to predict protein-binding sites, and V1.1.2 AutoDockVina was used for docking. The analysis on molecular docking refers to Δ Gbind, which is <−5 kJ.mol-1, indicating that the compound binds with the target to some extent.^[19]^ Finally, the docking results of compounds and proteins were analyzed and observed with PyMOL software based on Δ Gbind and binding time. The docking effect is better if the Δ Gbind is lower.

## 3. Result

Active ingredients of RX-MY and corresponding target protein information were screened with the TCMSP, the RX-MY compounds were 127 and 276 respectively. Shown by OB and DL’ features, 8 bioactive components of frankincense and 45 bioactive components of myrrh were selected, and 53 bioactive components were obtained (Table 1). These 2 drugs contain a variety of effective ingredients, significant for the multiple target effects of TCM.

**Table 1:** Active components network.

Mol No.	Name	OB (%)	DL	Herb
MOL001215	tirucallol	42.12	0.75	RX
MOL001241	O-acetyl-α-boswellic acid	42.73	0.7	RX
MOL001243	3alpha-Hydroxy-olean-12-en-24-oic-acid	39.32	0.75	RX
MOL001255	Boswellic acid	39.55	0.75	RX
MOL001263	3-oxo-tirucallic, acid	42.86	0.81	RX
MOL001265	acetyl-alpha-boswellic,acid	42.73	0.7	RX
MOL001272	incensole	45.59	0.22	RX
MOL001295	phyllocladene	33.4	0.27	RX
MOL001001	quercetin-3-O-β-D-glucuronide	30.66	0.74	MY
MOL001002	ellagic acid	43.06	0.43	MY
MOL001004	pelargonidin	37.99	0.21	MY
MOL001006	poriferasta-7,22E-dien-3beta-ol	42.98	0.76	MY
MOL001009	guggulsterol-VI	54.72	0.43	MY
MOL001013	mansumbinoic acid	48.1	0.32	MY
MOL001019	(7S,8R,9S,10R,13S,14S,17Z)-17-ethylidene-7-hydroxy-10,13-dimethyl-1,2,6,7,8,9,11,12,14,15-decahydrocyclopenta [a] phenanthrene-3,16-dione	35.75	0.48	MY
MOL001026	myrrhanol C	39.96	0.58	MY
MOL001027	myrrhanone A	40.25	0.63	MY
MOL001028	(8R)-3-oxo-8-hydroxy-polypoda -13E,17E,21-triene	44.83	0.59	MY
MOL001029	myrrhanones B	34.39	0.67	MY
MOL001031	epimansumbinol	61.81	0.4	MY
MOL001033	diayangambin	63.84	0.81	MY
MOL001040	(2R)-5,7-dihydroxy-2-(4-hydroxyphenyl) chroman-4-one	42.36	0.21	MY
MOL001045	(13E,17E,21E)-8-hydroxypolypodo-13,17,21-trien-3-one	44.34	0.58	MY
MOL001046	(13E,17E,21E)-polypodo-13,17,21-triene-3,18-diol	39.96	0.58	MY
MOL001049	16-hydroperoxymansumbin-13(17)-en-3β-ol	41.05	0.49	MY
MOL001052	mansumbin-13(17)-en- 3,16-dione	41.78	0.45	MY
MOL001061	(16S, 20R)-dihydroxydammar-24-en-3-one	37.34	0.78	MY
MOL001062	15α-hydroxymansumbinone	37.51	0.44	MY
MOL001063	28-acetoxy-15α-hydroxymansumbinone	41.85	0.67	MY
MOL001069	3β-acetoxy-16β,20(R)-dihydroxydammar-24-ene	38.72	0.81	MY
MOL001088	1α-acetoxy-9,19-cyclolanost-24-en-3β-ol	44.4	0.78	MY
MOL001092	[(3R,5R,8R,9R,10R,13R,14R,17S)-17-[(2S,5S)-5-(2-hydroxypropan-2-yl)-2-methyloxolan-2-yl]-4,4,8,10,14-pentamethyl-2,3,5,6,7,9,11,12,13,15,16,17-dodecahydro-1H-cyclopenta[a]phenanthren-3-yl] acetate	33.07	0.8	MY
MOL001093	cabraleone	36.21	0.82	MY
MOL001095	isofouquierone	40.95	0.78	MY
MOL001126	[(5aS,8aR,9R)-8-oxo-9-(3,4,5-trimethoxyphenyl)-5,5a,6,9-tetrahydroisobenzofurano[6,5-f][1,3]benzodioxol-8a-yl] acetate	44.08	0.9	MY
MOL001131	phellamurin_qt	56.6	0.39	MY
MOL001138	(3R,20S)-3,20-dihydroxydammar- 24-ene	37.49	0.75	MY
MOL001145	(20S)-3β-acetoxy-12β,16β,25-tetrahydroxydammar-23-ene	34.89	0.82	MY
MOL001146	(20S)-3β,12β,16β,25-pentahydroxydammar-23-ene	37.94	0.75	MY
MOL001148	3β- hydroxydammar-24-ene	40.27	0.82	MY
MOL001156	3-methoxyfuranoguaia-9- en-8-one	35.15	0.18	MY
MOL001164	[(5S,6R,8R,9Z)-8-methoxy-3,6,10-trimethyl-4-oxo-6,7,8,11-tetrahydro-5H-cyclodeca[b]furan-5-yl] acetate	34.76	0.25	MY
MOL001175	Guggulsterone	42.45	0.44	MY
MOL000358	beta-sitosterol	36.91	0.75	MY
MOL000449	Stigmasterol	43.83	0.76	MY
MOL000490	petunidin	30.05	0.31	MY
MOL000979	2-methoxyfuranoguaia-9-ene-8-one	66.18	0.18	MY
MOL000988	4,17(20)-(cis)-pregnadiene-3,16-dione	51.42	0.48	MY
MOL000996	Guggulsterol IV	33.59	0.74	MY

DL = drug-likeness, MY = Moyao, OB = oral bioavailability, RX = Ruxiang.

**Figure 2.**
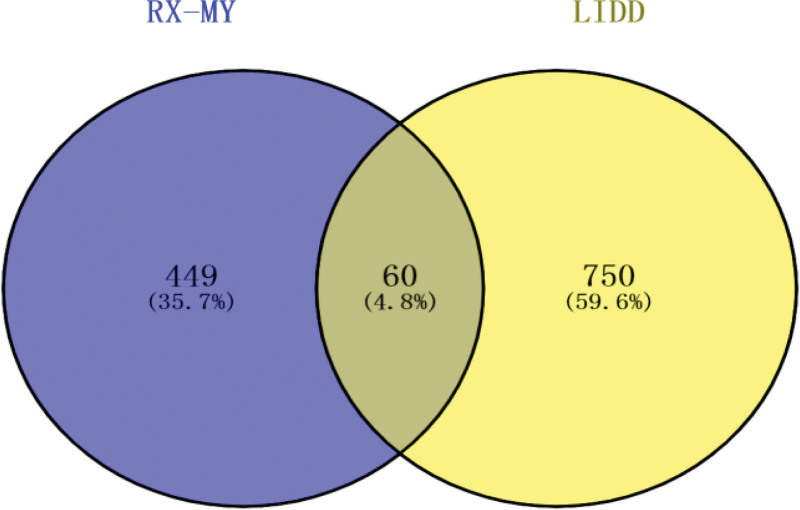
RX-MY Venn diagram and target for treating LIDD. LIDD = lumbar intervertebral disc degeneration, MY = Moyao, RX = Ruxiang

RX-MY and the target prediction of lumbar degenerative diseases by searching 53 active ingredients, 449 “RX-MY” targets were found. Totaling 854 targets on LDD were collected with GeneCards and DisGeNET databases, and 750 targets related to LDD were obtained after weight removal. After matching the potential targets of RX-MY with the LIDD-related targets, 60 overlapping targets were acquired and considered as the molecular basis of RX-MY against LIDD (Fig. 2).

**Figure 3.**
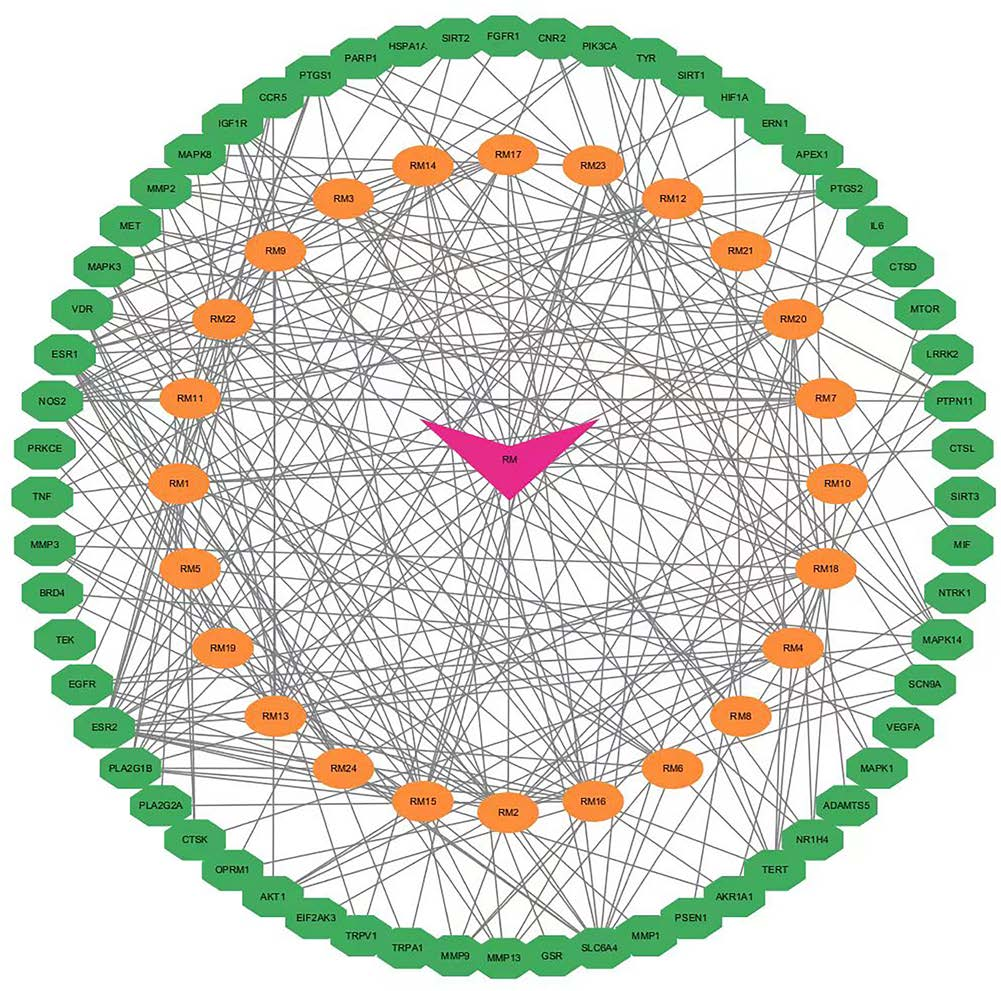
Network of active components-targets.

The network of “medicinal material and active component-target” was processed with Cytascape 3.9.1 (Fig. 3), which show totaling 55 nodes and 197 edges. A node means the active compound of the herb, an edge means active compound and the target protein correlation, and a link means the interaction between them.

**Figure 4.**
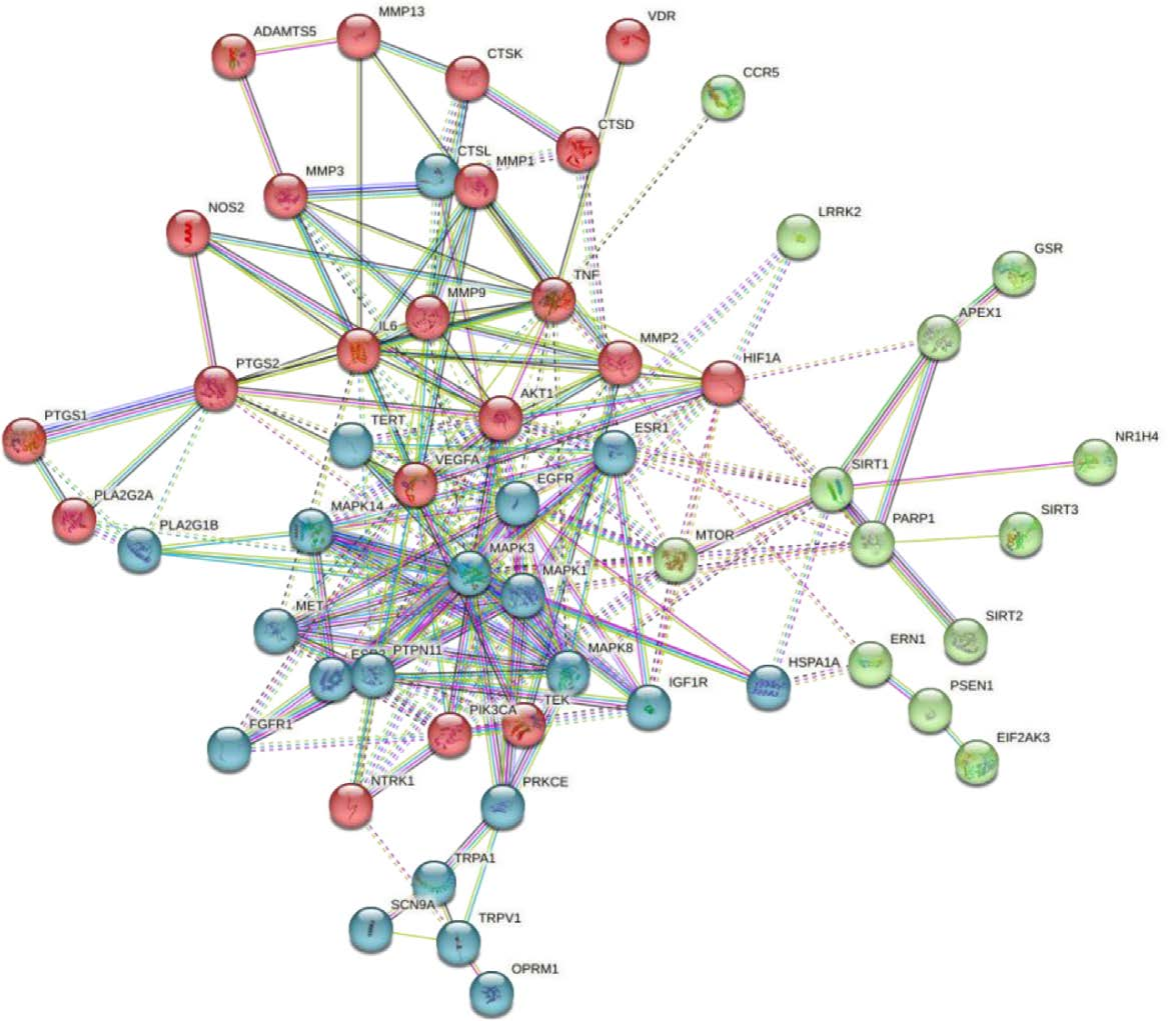
PPI network of targets for RX-MY against LIDD. LIDD = lumbar intervertebral disc degeneration, MY = Moyao, PPI = Protein-Protein Interaction, RX = Ruxiang.

**Figure 5.**
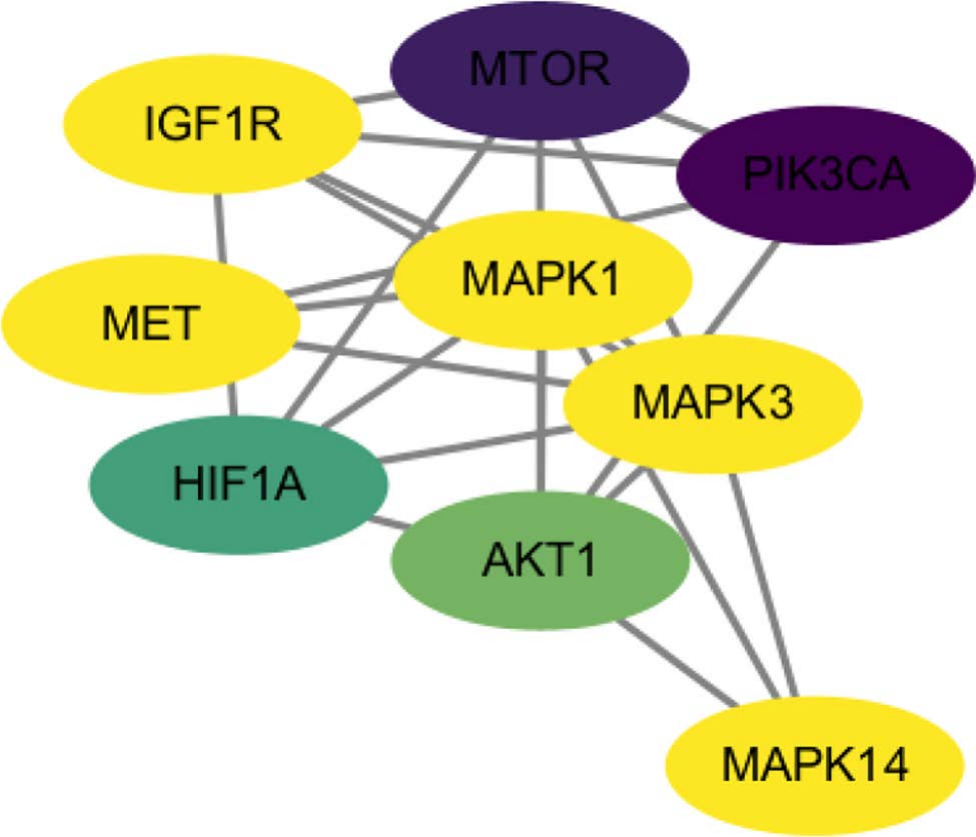
The cluster generated from PPI network. PPI = Protein-Protein Interaction.

The interaction between 60 overlapping genes and common target proteins was retrieved with String, and the PPI network of LIDD treated with “RX-MY” was constructed. The network (Fig. 4) consists of 54 nodes, 209 edges and an average node degree of 7.74. The tsv file was sourced from STRING. Cytoscape v3.9.1 was used for analyzing PPI network based on the tsv file. According to the criteria of DC, BC, CC and EC (≥ 7.27, ≥ 82.48, ≥ 0.41, and ≥ 0.10 respectively), 12 key targets were identified (Table 2), including 5 targets: MAPK3, EGFR, VEGFA, MAPK1 and AKT1 with a high degree value. The cluster. viz plug-in was further used to cluster the PPI network to obtain a subnetwork with higher connectivity (Fig. 5).

**Table 2:** 12 key targets RX-MY for treating LIDD.

Symbols	DC	EC	BC	CC
MAPK3	25	0.31397417	416.95993	0.5955056
EGFR	23	0.310428	272.78268	0.57608694
VEGFA	20	0.27029124	160.63171	0.5463917
MAPK1	20	0.24341309	259.92865	0.5520833
AKT1	19	0.27514517	148.89455	0.53535354
TNF	18	0.21506958	488.2413	0.53
IL6	17	0.24167977	119.85861	0.5145631
HIF1A	16	0.22790863	243.78325	0.5247525
ESR1	16	0.21403229	138.74431	0.48623854
MMP9	14	0.19084463	168.758	0.51960784
MAPK8	11	0.15382951	101.79822	0.50476193
PTGS2	11	0.15392959	107.994446	0.4732143

BC = betweenness centrality, CC = closeness centrality, DC = degree centrality, EC = eigenvector centrality.

**Figure 6.**
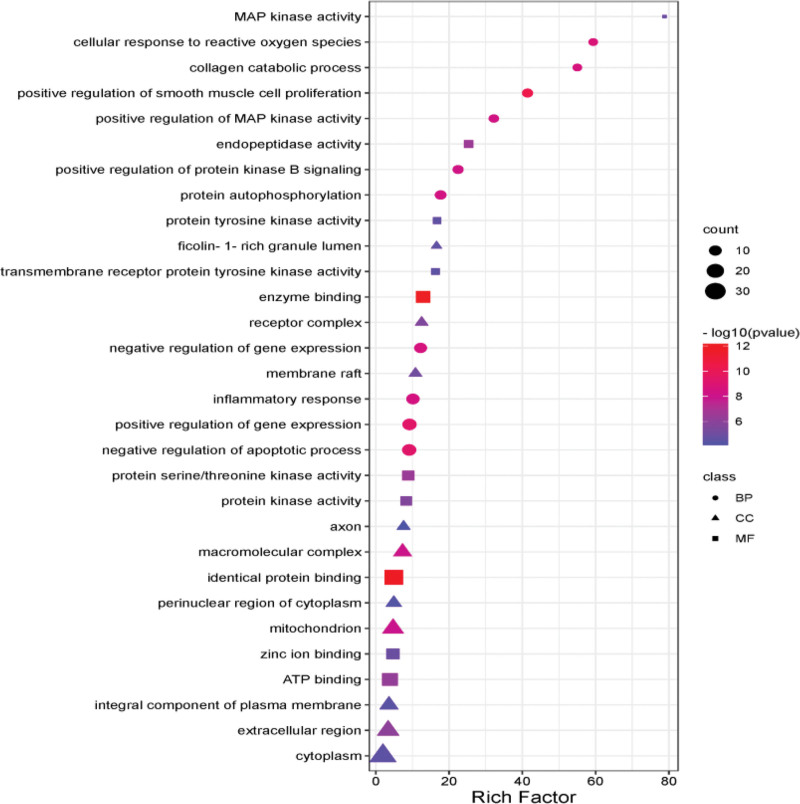
GO enrichment of the anti-LIDD effect of RX-MY. GO = gene ontology, LIDD = lumbar intervertebral disc degeneration, MY = Moyao, RX = Ruxiang.

GO enrichment of 60 potential targets for treating lumbar degenerative diseases with frankincense and myrrh was analyzed (*P* < .01) with DAVID, obtaining totaling 495 entries, including 365 BP, 60 CC, and 70 MP, and filtering out the first 10 entries of each part based on *P* value (Fig. 6). Multiple cell components and biological processes may also be involved based on the analysis results of GO enrichment, such as increase signaling of protein kinase B, activities of cytoplasm, protein serine/ kinases of threonine, protein tyrosine, transmembrane receptor protein tyrosine, inflammatory response, and decrease the apoptotic process.

**Figure 7.**
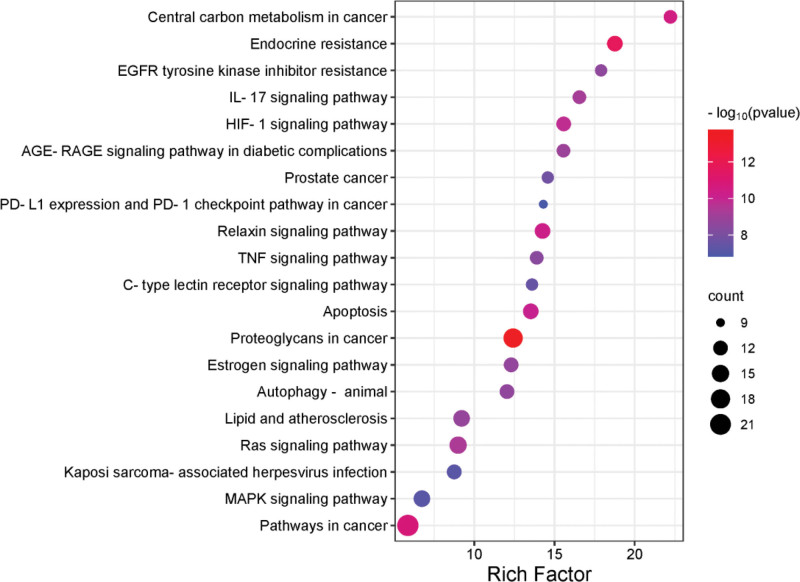
Enrichment of KEGG pathway of the anti-LIDD effect of RX-MY. KEGG = Kyoto Encyclopedia of Genes and Genomes, LIDD = lumbar intervertebral disc degeneration, MY = Moyao, RX = Ruxiang.

Through analyzing KEGG enrichment, 135 related signaling pathways (*P* < .01) were obtained in all. The bubble diagram of related signaling pathways (Fig. 7) made according to the results of *P* value showed that RX-MY treatment LIDD included the signaling pathways of HIF-1, IL-17, TNF, and MAPK. The signaling pathway of MAPK is involved in regulating apoptosis, proliferation, DNA transcription and translation, and in the regulation of oxidative stress and inflammatory response.^[20]^ The MAPK family is mainly composed of 3 subgroups: ERK, c-JNK and p38 MAPK. P38 MAPK significant for MAPK pathway, regulating inflammation and significant for bone metabolism,^[21]^ and it is closely related to IVDD. On the combination of the core target in cluster analysis diagram of the PPI network and the enrichment results of KEGG pathway, our research group predicted that frankincense-myrrh might mainly treat LIDD by inhibiting MAPK signal pathway.

**Figure 8.**
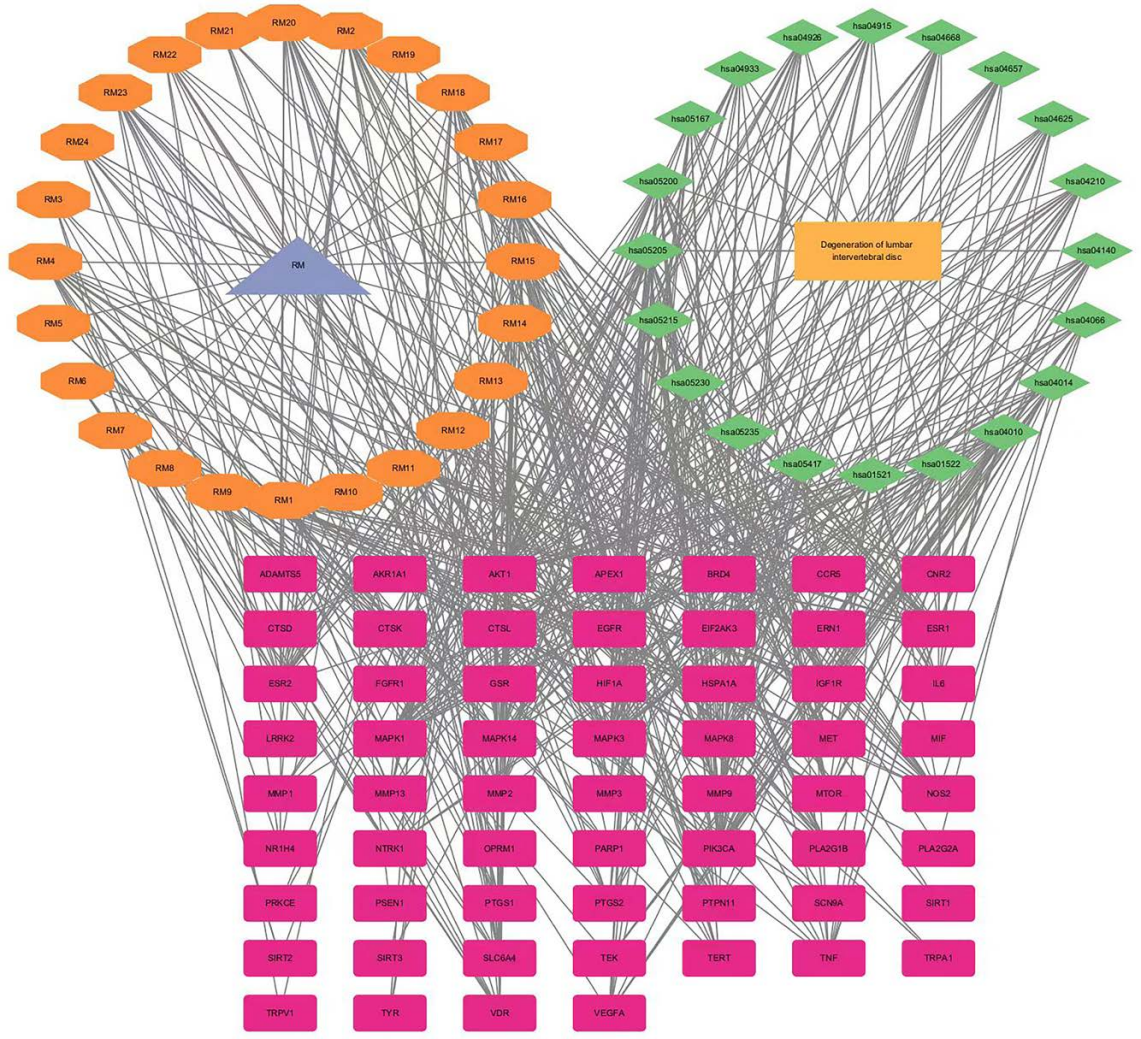
Compound–target–pathway of treating LIDD network. LIDD = lumbar intervertebral disc degeneration.

The network of “TCM - active ingredients - intersection target - signal pathway - disease” is diagramed with Cytascape 3.9.1 software, including 55 nodes and 197 edges. The yellow rectangle represents diseases, the green diamond represents signal pathways (20), the light purple triangle represents frankincense-myrrh, the orange polygon means the active components of frankincense-myrrh (24), and the crimson rectangle means key targets (60), including inflammation resistance, analgesic targets, etc, reflecting the characteristics of RX-MY intervention in LIDD through multicomponent, multitarget and, multi-channel methods (Fig. 8).

Molecules were docked with AutoDockVina1.1.2 to deeply verify the active compound and the key target binding force and increase the target network accuracy. Analysis on docking the 5 highest targets of DC (MAPK3, EGFR, VEGFA, MAPK1, AKT1) and 5 compounds were selected according to DC (3-oxy-tirucalic, acid, isofouquierone, (7S, 8R, 9S, 10R, 13S, 14S, 17Z)- 17-ethylidene-7-hydroxy-10,13-dimethyl-1,2,6,7,8,9,11,12,14,15-decahydrocyclopenta [a] phenanthrene-3,16-dione [(5S, 6R, 8R, 9Z)- 8-methoxy-3,6,10-trimethyl-4-oxo-6,7,8,11-tetrahydro-5H cyclodeca [b] furan-5-yl] acetate and pelargonidin), as shown in in Table 3. Their Δ Gbind was mostly < - 5kJ · mol—1 with high binding force.

**Table 3:**
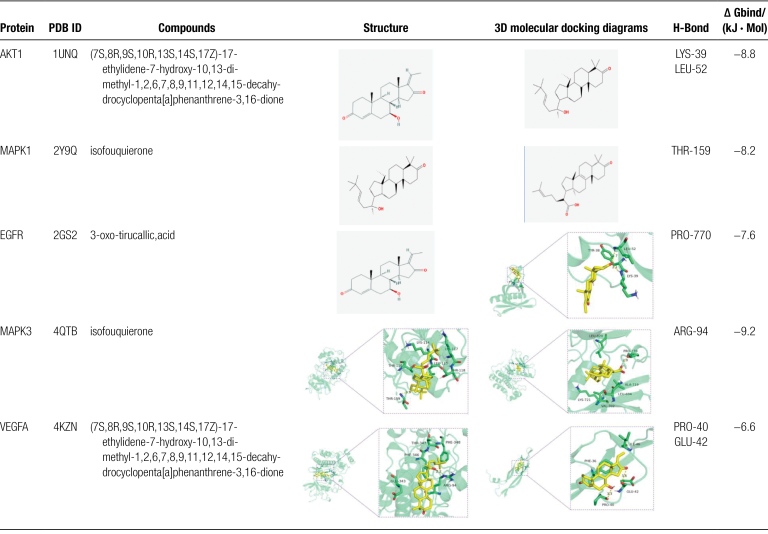
Active compound score of key targets of RX-MY.

## 4. Discussion

LIDD mainly reflects pain in low back and legs. Its pathogenesis in previous studies was mostly about regulating inflammation and inhibiting the inflammatory mediator of lumbar degenerative disease. Various inflammatory factors interact with each other through multiple cell signal pathways to induce inflammatory cell infiltration, form a complex inflammatory network and produce cascade reactions. After lumbar degenerative disease, it not only promotes the occurrence of degeneration, but also participates in the self-repair process after injury. Therefore, the formation of lumbar degenerative disease is caused by several cytokines or pathways. The treatment of LIDD in TCM mainly focuses on tonifying the kidney qi, promoting blood circulation and stasis removal. Frankincense-myrrh is the main manifestation of the TCM clinical “blood circulation promotion and blood stasis removal, regulating qi and relieving pain.” According to modern pharmacological studies, frankincense-myrrh is not simple superposition of the pharmacological effects but actively resisting inflammation, anticancer, analgesic, antibacterial and promoting blood circulation together.^[11]^

Network pharmacology was applied herein for predicting and clarifying the potential molecular mechanism underlying the effect of RX-MY on LIDD. The active ingredient target network of frankincense-myrrh obtained 60 target genes with the influence of 24 bioactive compounds in frankincense-myrrh. Five compounds of HLJDD were identified as potential active, including oxo-tirucallic, acid, isofouquieron, (7S,8R,9S,10R,13S,14S,17Z)-17-ethylidene-7-hydroxy-10,13-dimethyl-1,2, 6,7,8,9,11,12,14,15-decahydrocyclopenta[a]phenanthrene-3,16-dione, [(5S,6R,8R,9Z)-8-methoxy-3,6,10-trimethyl-4-oxo-6,7,8,11-tetrahydro-5H -cyclodeca [b]furan-5-yl] acetate, pelargonidin. Among them, 3-oxo-tirucalic, acid is mastic acid, and mastic acid, as the main bioactive component of mastic, has a strong inflammation resistance effect. Through the regulation of mastic acid (Bas) on pathways of inflammatory cytokines and protein kinase. Liang et al studied combining AKBA (3) and As2O3 to inhibit the secretions of MMP-1, MMP-2, MMP-9, TNF-α, and IL-β.^[22]^ The adjuvant-induced arthritis rats were treated with 0.90 g·kg^−1^ frankincense extract daily for 10 days. According to the results, the frankincense extract has significant inflammation resistance effects, which may be caused by the inhibition of proinflammatory cytokines.^[23]^ Myrrh is mainly composed of sesquiterpenoids, which have strong inflammation resistance and analgesic effects. Modern research has found that sesquiterpenoids related compounds in myrrh has analgesic properties, which are blocked by morphine antagonist naloxone, acting on opioid receptors in the nervous centralis^[24]^; Myrrh has also been found to be an alternative drug for further treating neuralgia^[25]^; According to the study of Germano et al, when male volunteers took 400 mg myrrh compound daily for 20 days continuously, most of all pain conditions were relieved. Female volunteers only took 200 mg daily for 20 days, whose pain caused by low back pain and fever could significantly relieve.^[26]^ However, note that frankincense and myrrh have different proportions and contents of terpene compounds and are essential as one of their main active ingredients before and after combining, thus affecting their pharmacological effects and curative effects.^[27,28]^ This effect is manifested in increasing the synergetic effect of pharmacodynamics, including synergetic inflammation resistance, synergetic analgesia, and synergetic blood circulation.^[29,30]^

PPI network analysis identified 60 targets of RX-MY acting on LIDD. “RX-MY” in the treatment of LIDD, MAPK3 mainly target on AKT1, MAPK3, EGFR, VEGFA and MAPK1. MAPK1 plays a significant role in MAPK signal pathway, which participates in various stress reactions, such as inflammation and proliferation, differentiation, transcription, regulation and development of cells, and are closely related to neuropathic pain.^[31]^ AKT1 protein participates in multiple biological processes, such as metabolism, proliferation, cell survival, growth, insulin signal transduction, and angiogenesis. The key targets predicted by this study can be considered as one of the future research directions for the treatment of lumbar degenerative diseases.

The GO enrichment results show that RX-MY acts on the core target of LIDD and affects biological processes, including decreases apoptotic process and cellular response, and activates oxygen specifications, inflammatory response, protein autophysiology, and increase the activity of MAP kinase, etc. These biological processes may be significant for LIDD to occur and develop. Through analyzing the enrichment of KEGG pathway, the signaling pathways of HIF-1, TNF, MAPK, etc may be potential for RX-MY treatment of LIDD. MAPK signal pathway regulates apoptosis, proliferation, DNA transcription and translation, and in the regulation of oxidative stress and inflammatory response, which exists widely in mammalian cells. MAPK family is mainly composed of 3 subfamilies of ERK, c-JNK and p38 MAPK. ERK is responsible for transmitting the signal of cell membrane surface receptors into the cell. JNK cooperates with ERK to regulate cell proliferation and differentiation, morphology maintenance and cytoskeleton construction; it is also a significant signaling pathway in bone metabolism.^[20]^ P38MAPK is the key factor in regulating inflammatory response in MAPK pathway. When external signals stimulate cells, including growth factors and cytokines as well as cell stress, it will activate the MAPK pathway, and this series of reactions begins from Ser/Thr residue of MAPK kinase (MAPKKs), which is phosphorylated and activated by MAPKKs, successively activating the adjacent residues of threonine and tyrosine in the conserved ThrX-Tyr motif of the MAPKs activation ring, and X corresponds to glutamate in ERK Proline in JNK or glycine in p38.^[32]^ P38MAPK pathway is closely related to IVDD. Zhu et al^[33]^ simulated intervertebral disc herniation found that p38MAPK pathway was activated in rats’ lumber disc tissue, and the level of apoptosis and caspase-3 in nucleus pulposus increased. After blocking p38MAPK pathway with p38i (p38MAPK inhibitor), the level of apoptosis and caspase-3 decreased significantly, suggesting that p38MAPK pathway may regulate cell apoptosis in reabsorbing herniated tissues. According to the dock results, most of the 5 main active components and the 5 core targets were high, including (7S, 8R, 9S, 10R, 13S, 14S, 17Z)-17-ethylidene-7-hydroxy-10,13-dimethyl-1,2,6,7,8,9,11, 12,14,15-decahydrocyclopenta [a] phenanthrene-3,16-dione powerfully binds with AKT1 and VEGFA, isofouquierone powerfully binds with MAPK1 and MAPK3, while 3-oxo-tirulone callic, Acid powerfully binds with EGFR, which is mainly be targeted on treating LIDD with RX-MY.

## 5. Conclusion

Thus, the network pharmacological analysis provides many verifiable hypotheses for the molecular mechanism of RX-MY against LIDD, and also predicts (7S,8R,9S,10R,13S,14S,17Z)-17-ethylidene-7-hydroxy-10,13-dimethyl-1,2,6,7,8,9,11,12,14,15-decahydrocyclopenta[a]phenanthrene-3,16-dione, isofouquierone, 3-oxo-tirucallic, acid which will be the potential active component of RX-MY, and acts on key genes and regulates a variety of biological processes and pathways. Due to the change of components after combining frankincense and myrrh, it is impossible to separate chemical components and screen simple active substances for study, but this network pharmacological prediction can still provide a theoretical explanation for the treatment of LIDD by RX-MY. We will combine animal and molecular experiments in our next study to clarify the material basis of RX-MY, as well as, its mechanism of action in treating LIDD.

## Author contributions

**Conceptualization:** Yun Lu, Yu Hu, Xinghua Song.

**Data curation:** Yun Lu, Haopeng Luan, Cong Peng.

**Formal analysis:** Yun Lu, Junjie Ma.

**Funding acquisition:** Yu Hu.

**Methodology:** Haopeng Luan, Cong Peng, Zhe Li.

**Software:** Yun Lu, Junjie Ma.

**Writing – original draft:** Yun Lu, Haopeng Luan, Cong Peng.

**Writing – review & editing:** Yun Lu, Yu Hu, Xinghua Song.
